# Phage-derived endolysin prevents dissemination of methicillin resistant *Staphylococcus aureus* during oral co-infection with *Candida albicans*

**DOI:** 10.1128/aac.01812-25

**Published:** 2026-06-15

**Authors:** Katrien Van Dyck, Michel A. Hoogenkamp, Fritz Eichenseher, Patrick Van Dijck, Bastiaan P. Krom

**Affiliations:** 1 Laboratory of Molecular Cell Biology, Department of Biology, Institute of Botany and Microbiology, KU Leuven 26657, Leuven, Belgium; 2 KU Leuven One Health Institute, Leuven, Belgium; 3 Department of Preventive Dentistry, Academic Centre for Dentistry Amsterdam, University of Amsterdam and Vrije Universiteit Amsterdam 1192, Amsterdam, the Netherlands; 4 Micreos GmbH, Wädenswil, Switzerland; The Peter Doherty Institute for Infection and Immunity, Melbourne, Victoria, Australia

**Keywords:** *Candida albicans*, bloodstream infection, oral co-infection, MRSA

## Abstract

The oral cavity is colonized by many species of bacteria and fungi without causing serious infections of hosts. However, upon loss of homeostasis, some of these colonizers have the potential to invade the host and cause life-threatening infections. The fungus *Candida albicans* and the gram-positive bacterium *Staphylococcus aureus* represent well-studied examples. In immunocompromised mice, oral co-colonization with these two species can result in dissemination of *S. aureus* through the body, causing *S. aureus* bacteremia and subsequent sepsis. In the proof-of-principle experiments described here, we show effective prevention of dissemination of methicillin-resistant *S. aureus* (MRSA) using an innovative endolysin-derived chimeric protein, XZ.700. Using the established murine model for oral co-colonization, we evaluated the effect of treatment with XZ.700 in drinking water (*ad libitum*) and applied as a gel formulation twice a day. XZ.700 did not show any adverse reaction in the mice treated and did not show a reduction of *C. albicans* colonization. XZ.700 in drinking water completely prevented detectable dissemination of MRSA in co-infected mice and significantly reduced weight loss. The gel formulation showed a trend in decreased MRSA colonization of the tongue and dissemination to the kidneys, although this was not significantly different from the placebo control. Taken together, the results presented here indicate that selective removal of MRSA from the oral cavity of mice with XZ.700 during co-infection with *C. albicans* is effective in preventing dissemination. Further studies need to show clinical applicability of XZ.700 and related compounds in prevention of sepsis in at-risk patients.

## INTRODUCTION

The oral cavity is colonized by a range of bacterial and fungal species (1). While oral diseases (e.g. caries and gingivitis) are common, the oral mucosa represents an effective barrier against microbial invasion of the host (2). The tongue represents a unique oral mucosal niche as it contains crevices protecting the microbes that colonize the tongue against swallowing. In addition, although oral hygiene measures aimed at tongue cleaning are available, its use is not as common as tooth brushing, resulting in the presence of mature, stable biofilms on the tongue surface. In a healthy situation, a homeostatic relationship between the tongue microbiota and the host is maintained (3).

A common colonizer of the tongue is the polymorphic fungus *Candida albicans* (4). This opportunistic pathogen is commonly present in the oral cavity of healthy individuals, where it is considered a commensal (5). Its presence influences the microbiome (6), and its role in homeostasis as a keystone commensal has been proposed (7). When the balance between the host and the microbiome is lost, *C. albicans* can cause disease, often related to increased colonization and morphological switching from yeast to hyphal morphology (8). Loss of homeostasis (i.e., dysbiosis) can be the result of changes in the microbiota (e.g., upon antibiotic use) or of changes in the host’s immune response due to development and treatment of underlying diseases (e.g. rheumatoid arthritis and diabetes) (9). In addition, dysbiosis can develop during aging (reviewed in reference 10). Oral candidiasis (oropharyngeal candidiasis [OPC]), caused by invasion of hyphae into the epithelium of the oral mucosa, is a common condition among very young and elderly individuals and immunocompromised patients, such as those infected with HIV. Irrespective of the reason, OPC commonly remains localized to the oral mucosa and rarely becomes life-threatening.

*C. albicans* coexists with *Staphylococcus aureus* in several host niches, and it is widely recognized that they interact synergistically (11, 12). Recently, we and others reported on a new complication related to OPC (13–15). During OPC, invasion of the mucosal surface by hyphae of *C. albicans* in the presence of bacteria can result in disseminated bacterial disease, i.e., bacteremia, potentially leading to sepsis (15). This was convincingly shown in a series of studies using a murine model of oral co-colonization with *C. albicans* and *S. aureus* (9, 13, 14). Hyphae of *C. albicans* are recognized by *S. aureus* through interaction with hypha-specific cell wall proteins Als3p (13, 14) and Als1p (16). When bacteria-covered hyphae invade the mucosal lining, host phagocytes are attracted by the presence of the fungus (15). However, while the hyphae are too large to be phagocytosed by the immune cells, adhering *S. aureus* are readily phagocytosed and transported by the phagocyte to the cervical lymph nodes (15). *S. aureus* has the ability to survive within phagocytes, where they could also be protected from antibiotics, resulting in long-term survival of phagocytosed bacteria (17, 18). Isolation of viable *S. aureus* from cervical lymph nodes indicates that they can disseminate through the bloodstream, illustrated by isolation of viable *S. aureus* from the kidneys (15, 16).

Bloodstream infection (bacteremia) and consequently sepsis with *S. aureus* represent a life-threatening infection requiring urgent treatment with antibiotics. However, adhesion and biofilm formation of *S. aureus* on *C. albicans* directly impact the efficacy of antibiotic treatment (19–21). Additionally, methicillin-resistant *S. aureus* (MRSA) is particularly common in care facilities (22) and often isolated from the tongue, limiting the antibiotics available for treatment. Taken together, there is an urgent need for preventive strategies that are not based on antibiotics.

Phages have long been recognized as a promising alternative for antibiotics (23), yet the application of phages into common practice has remained disappointingly limited. Alternatively, phage-derived proteins, most notably phage-encoded endolysins, are on the brink of finding their way to clinical use (24). Endolysins are cell wall-hydrolyzing proteins that specifically degrade the peptidoglycans in the cell wall of bacteria and have, therefore, gained attention as a novel class of antibacterial agents (25). We recently showed that a chimeric endolysin, XZ.700, consisting of parts of *S. aureus* bacteriophage endolysin Ply2638 (26) and lysostaphin (27), was effective in killing MRSA biofilms (28). The enzymatic cleavage of the staphylococcal cell wall by XZ.700 is dependent on both the recognition of the cell wall by a cell wall-binding domain (with SH3b homology) and by the specific hydrolytic activity of the two enzymatically active domains (an amidase and an endopeptidase) (17, 18). XZ.700 is produced as a recombinant protein in a microbial expression system (29).

In the presence of *C. albicans*, *S. aureus* is less sensitive to antibiotic treatment (19, 20). Use of antibiotics such as vancomycin, to prevent bacterial dissemination upon oral co-colonization, is, therefore, less likely to be successful. In addition, antibiotic-resistant strains of *S. aureus* are common. In this study, we set out to determine if XZ.700 endolysin-based treatment could be a promising avenue to prevent *S. aureus* dissemination upon OPC. We use MRSA to mimic a worst-case scenario, infection with antibiotic-resistant *S. aureus*. We hypothesized that endolysin-based therapy, using XZ.700, can kill *C. albicans*-adhered MRSA and, thus, prevent dissemination in an established murine model of oral co-colonization by *C. albicans* and MRSA. We, therefore, initially investigated the efficacy of the chimeric endolysin XZ.700 on MRSA adhering to hyphae of *C. albicans*. Subsequently, we determined the efficacy in the complexity of the oral environment, i.e., in presence of human saliva. Finally, we evaluated two clinical preventative approaches, one by providing XZ.700 in water and the other in a gel, using an established murine model for oral co-colonization of *C. albicans* and MRSA.

## RESULTS

**Fig 1:**
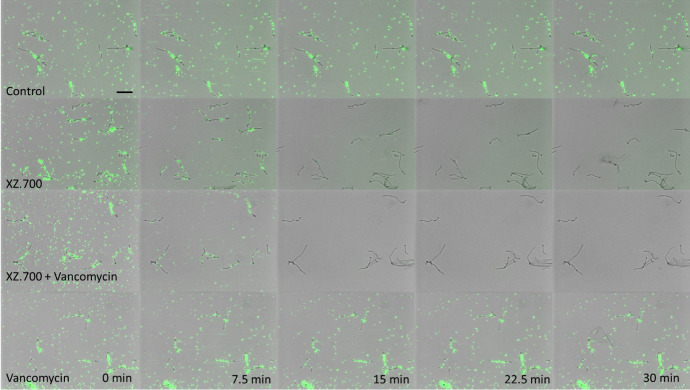
XZ.700 treatment, but not vancomycin treatment, removes GFP-expressing MRSA attached from the hyphae of *C. albicans***.** Panel A shows overlays (every 7.5 min) made of representative time-lapse images combining the brightfield and fluorescein isothiocyanate (FITC) channels. XZ.700 was added at 25 µg/mL and vancomycin at 4 µg/mL. Control represents the carrier control. Images from the same experiment are shown; experiments included duplicate wells and were performed at least twice. The size bar indicates 20 µm.

Previous work has shown that *S. aureus* readily adheres to hyphae of *C. albicans* (13, 14). The effect of endolysins on adhering MRSA was evaluated by measuring loss of GFP fluorescence over time of GFP-labeled *S. aureus* USA300. Lysis by endolysins resulted in loss of the GFP signal within 5 min, while vancomycin treatment did not (Fig. S1). There was no antagonistic effect of vancomycin on XZ.700 activity. Using this system, we first focused on the ability of XZ.700 to remove GFP-expressing MRSA that had just adhered to the hyphae of *C. albicans*, i.e., 30 min after the start of adhesion. In the absence of XZ.700, all GFP-expressing MRSA remained visibly adhered on the hyphae of *C. albicans* over the time of the experiment (30 min) (Fig. 1). In contrast, treatment with XZ.700 resulted in visible loss of GFP-expressing MRSA from the hyphae of *C. albicans* within 7.5 min after the start of the treatment, with complete loss of visible GFP-expressing MRSA. A similar result was obtained when a treatment with XZ.700 and vancomycin was applied. In contrast, in the timeframe of this experiment, vancomycin treatment alone did not result in loss of fluorescence (Fig. 1).

**Fig 2:**
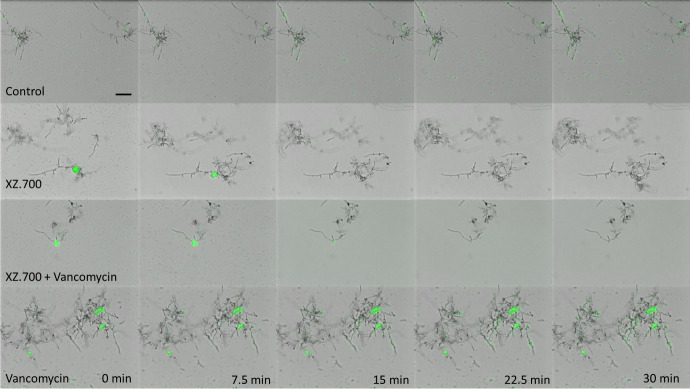
XZ.700 treatment, but not vancomycin treatment, removes GFP-expressing MRSA after growth for 3 h on hyphae of *C. albicans***.** Panel A shows an overlay (every 7.5 min) made of representative time-lapse images combining the brightfield and FITC channels. XZ.700 was added at 25 µg/mL and vancomycin at 4 µg/mL. Control represents the carrier control. Images from the same experiment are shown; experiments included duplicate wells and were performed at least twice. The size bar indicates 20 µm.

After initial adhesion, *S. aureus* will grow into a biofilm on the surface of *C. albicans* hyphae (20). Therefore, we evaluated the activity of XZ.700 against 3-h-old MRSA biofilms (representing young initial biofilms) on the surface of *C. albicans* (Fig. 2). Untreated and vancomycin-treated MRSA biofilms remain unchanged, while both XZ.700 and XZ.700 + vancomycin-treated biofilms again showed a loss in fluorescence, indicative of cell lysis.

**Fig 3:**
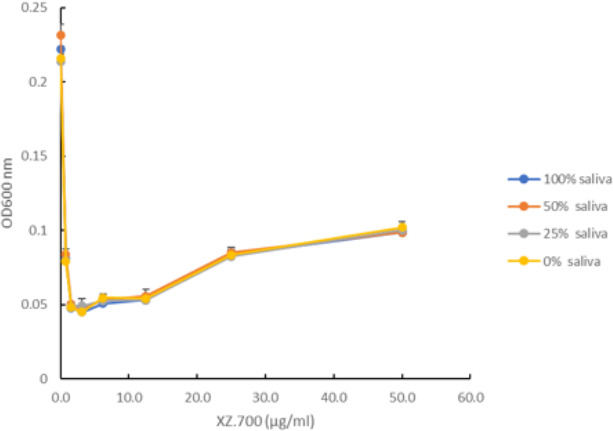
Effect of four different concentrations of human saliva on the lytic activity of XZ.700 against MRSA. Lysis was determined by measuring the optical density at 600 nm (OD_600_) of a standardized bacterial suspension (*n* = 2) containing *S. aureus* cells after 5 min. No difference was observed in concentration-dependent lysis of MRSA between the samples containing different concentrations saliva. Lines represent the lytic activity, as determined by a reduction in the optical density (OD_600_). Blue line, XZ.700 in the presence of 100% saliva; orange line, XZ.700 in the presence of 50% saliva; gray line, XZ.700 in the presence of 25% saliva; yellow line, XZ.700 in the presence of FBS:PBS.

In the oral cavity, saliva is always present. Saliva is a complex bodily fluid consisting of many different proteins, as well as electrolytes and microbes (30). We initially evaluated the effect of human saliva on the activity of XZ.700 against planktonic MRSA. Different concentrations of filtered human saliva in combination with different concentrations of XZ.700 indicated that there is no inhibition of lytic activity (Fig. 3). The highest lytic activity was observed using 25 µg/mL XZ.700, and lower levels of lytic activity were observed at higher concentrations.

Based on the *in vitro* data described above, it was decided that XZ.700 could potentially protect against dissemination of *S. aureus* (including MRSA) from the oral cavity upon co-colonization with *C. albicans*. We, therefore, designed two mouse studies to test the activity of XZ.700 using our murine model for oral co-colonization with *C. albicans* and *S. aureus*. The first study applied XZ.700 in drinking water of mice *ad libitum*. A pilot study with a small number of mice showed that XZ.700 does not affect the health and well-being of the mice (data not shown).

**Fig 4:**
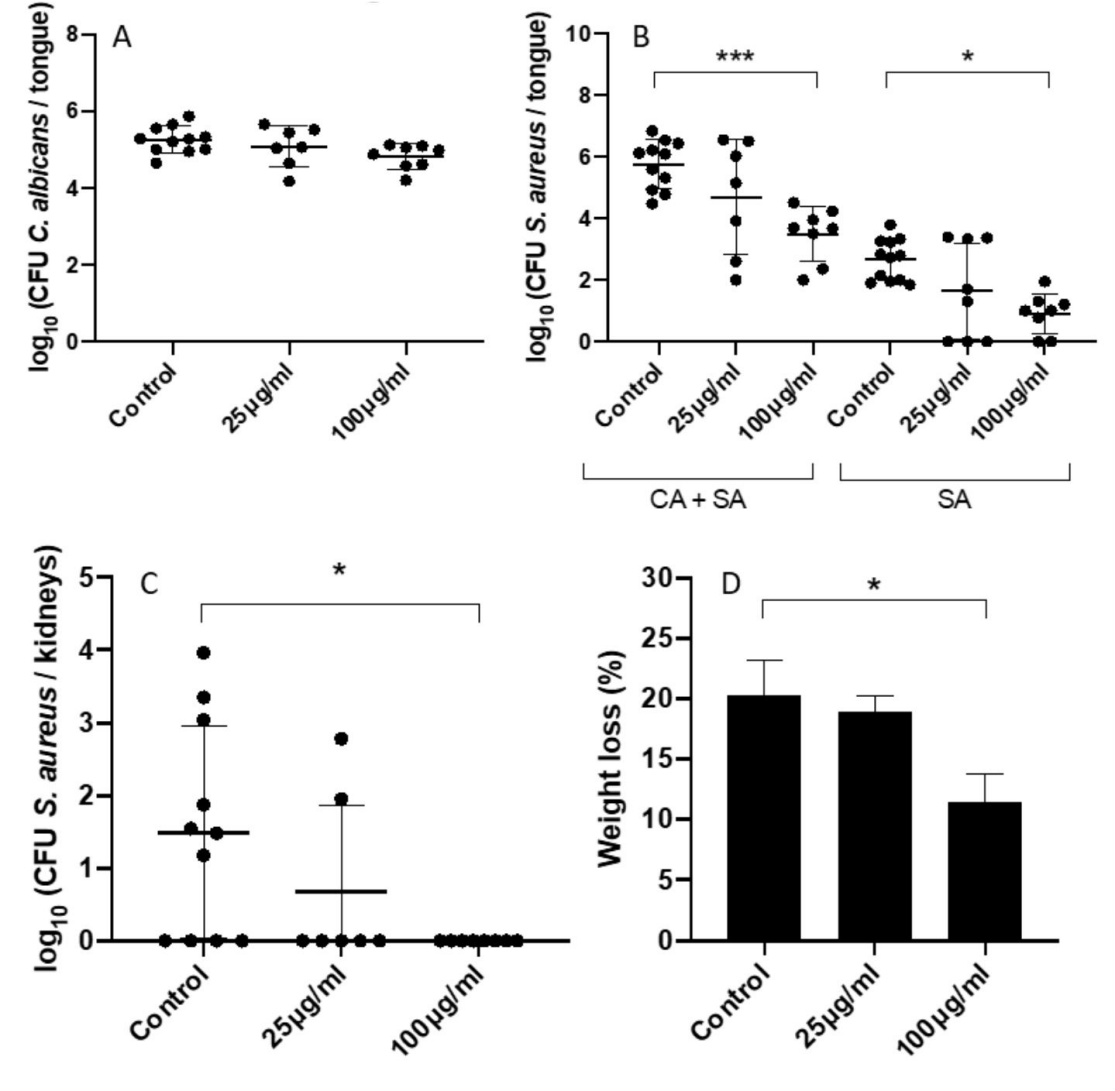
Effect of XZ.700 on the murine model of oral co-infection with *C. albicans* and MRSA. Groups of mice with induced oral candidiasis were inoculated with MRSA on the tongue. Colonization of the tongue (co-colonization) and of the kidney (dissemination) was determined by colony-forming unit (CFU) plating for *C. albicans* (**A**) and MRSA (**B** and **C**). Weight loss is expressed as % loss compared to pre-inoculation (**D**). Group sizes varied from *n* = 7 to *n* = 11, reflecting variability inherent to the infection model. Only if significance was found, these are shown in the figures and correspond to the Bonferroni-corrected *post hoc* tests (*, *P* < 0.05 and ***, *P* < 0.001 and ****, *P* < 0.0001).

XZ.700 in drinking water did not affect colonization of the tongue by *C. albicans* (Fig. 4A). In contrast, a significant decrease of approximately 2 log units was observed in colonization of the tongue by MRSA when XZ.700 was applied at 100 µg/mL (Fig. 4B). In the group with 100 µg/mL XZ.700, MRSA did not colonize the kidneys, in contrast to the untreated control (Fig. 4C). Weight loss, an objective measure for onset of sepsis, was significantly lower in the group treated with 100 µg/mL XZ.700 compared to the untreated group (Fig. 4D).

Fig. 4B shows results from two experimental conditions: mice infected with MRSA alone and mice co-infected with *C. albicans* and MRSA. This illustrates how treatment with different concentrations of the endolysin affected MRSA colonization in the context of single versus co-infection. Co-infected mice show higher levels of MRSA on the tongue compared to mono-infected mice, consistent with the known adhesion of *S. aureus* to C. *albicans*. However, significantly less MRSA was recovered from the tongues of mice treated with the highest concentration of XZ.700 (100 µg/ml), both for co-infected mice and mice only colonized with MRSA. Oral bacterial colonization was also slightly reduced for mice treated with the lower concentration of XZ.700 (25 µg/mL), yet this was not significant due to the large variation. This variation could be explained by the fact that XZ.700 was available in drinking water *ad libitum*.

**Fig 5:**
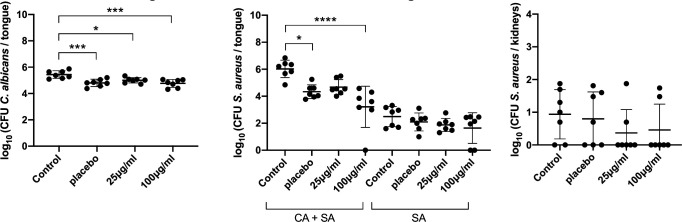
Colonization in mice receiving different gel-based treatments. The control group did not receive any treatment; the placebo group twice daily received 20 µL gel without XZ.700; the treatment groups twice daily received 20 µL gel containing 25 µg/mL or 100 µg/mL. Differences in group size reflect variability inherent to the infection model. Only if significance was found, these are shown in the figures and correspond to the Bonferroni-corrected *post hoc* tests (*, *P* < 0.05 and ***, *P* < 0.001 and ****, *P* < 0.0001). Total number of mice per group is indicated by the individual data points (*n* = 7 with one exception, where *n* = 6).

In the second study, XZ.700 was applied twice daily in a gel at 25 or 100 µg/mL concentration. Application of the gel alone (placebo) resulted in decreased colonization of the tongue with *C. albicans* and MRSA (Fig. 5). It was observed that after application of the gel, presence of the gel alone, or with XZ.700, as soon as the mice woke up, they started licking and swallowing to remove the gel. This could potentially remove chunks of the biofilm and thereby significant amounts of both *C. albicans* and MRSA cells, possibly explaining the difference between untreated and placebo groups. Alternatively, as there was no significant difference between treatment with XZ.700 or placebo gel, this could indicate that endolysin is less efficient in the gel formulation compared to in drinking water. This may be explained by the fact that mice were potentially more often exposed to the endolysin when it is administered in drinking water, compared to the twice-daily administration via the gel formulation. An additional decrease in MRSA colonization on the tongue after treatment with 100 µg/mL XZ.700-containing gel was observed; however, this decrease was not significant compared to the placebo treatment. Similarly, dissemination of MRSA to the kidney was decreased in the group treated with XZ.700-containing gel; however, this difference was not significant. Of note, in five of the seven mice, no dissemination could be determined, compared to two and three out of seven mice in the control and placebo groups, respectively, illustrating a protective effect.

## DISCUSSION

In the present study, we investigated the potential use of endolysin-based approaches to prevent dissemination of MRSA from the oral cavity upon co-colonization with *C. albicans*. While adhesion of *S. aureus* to the hyphae of *C. albicans* is related to decreased sensitivity to vancomycin (20), it did not prevent lysis by XZ.700. In addition, filter-sterilized human saliva still allowed lysis of GFP-expressing MRSA. When mixed-species MRSA-*C. albicans*-saliva biofilms were treated, loss of GFP fluorescence was also observed (data not shown); however, due to the nature of these complex biofilms combined with microscopy, it was not possible to quantify this effect. The active concentration of XZ.700 is below 25 µg/mL, with higher concentrations showing lower lysis (Fig. 3). This has been shown for XZ.700 before (28).

Using the established murine model for staphylococcal dissemination upon oral co-colonization with *C. albicans* (13–16), we showed that continuous availability of XZ.700 in drinking water reduced oral colonization with MRSA by approximately 100-fold and completely prevented subsequent dissemination of MRSA to the kidneys. Importantly, treatment with XZ.700 also reduced weight loss, an important clinical parameter of sepsis in the murine model. Combined with observations by the animal technician on the general health and well-being, this indicated that treatment of orally co-colonized mice with XZ.700 prevented symptoms of disease. Twice-daily application of XZ.700 in a gel reduced colonization of the tongue and dissemination; however, due to the small sample size, this reduction was not significant.

Oral co-colonization with *C. albicans* and *S. aureus* represents a novel porte d’entrée for staphylococci (13–16). MRSA is a common colonizer of the oral cavity, especially in care facilities (22). Since oral candidiasis is also common in patients of these care facilities, this mechanism of invasion could be more frequent than currently recognized. Specific decontamination of the mucosa is a general approach in care facilities, most notably to prevent bacterial sepsis. However, overuse of antibiotics is being scrutinized due to increased development of (multi)-resistant microorganisms. Applying a *S. aureus*-specific phage-derived endolysin, e.g., XZ.700, as a novel approach in prevention of bacteremia in at-risk patient groups could, therefore, represent an attractive alternative for antibiotics.

In the present study, we observed no toxic or allergenic reaction to XZ.700 in mice upon oral administration. This is not unexpected as phages and their lysins are common in the human microbiota, and the oral administration ensured inactivation of the endolysin in the low-pH environment of the stomach. Previous *in vitro* studies using human cell lines also did not show any cytotoxicity of XZ.700 (28). Taken together, the results presented here indicate that selective removal of *S. aureus* from the oral cavity during co-infection with *C. albicans* is effective in preventing bacterial dissemination. Further studies need to show the clinical applicability of XZ.700 and related compounds in prevention of sepsis in at-risk patients.

## MATERIALS AND METHODS

### Strains and growth conditions

*C. albicans* SC5314 (31) was routinely cultured on Tryptic Soy Agar or Yeast extract-Peptone-Dextrose (YPD) at 30°C, and when appropriate, precultures were grown in yeast nitrogen base containing 0.5% D-glucose, pH 7.0 (YNBG) at 30°C while shaking at 150 rpm. The MRSA reference strain *S. aureus* USA300-GFP (pMV158-GFP) was constructed previously (28) and grown routinely in Tryptic Soy Broth (TSB) at 37°C under aerobic conditions in the presence of 10 µg/mL tetracycline to maintain the fluorescence marker (32). Loss of GFP fluorescence in GFP-expressing bacteria is applicable to evaluate fast antimicrobial activity such as lysis due to endolysins (Fig. S1) and killing by disinfectants (33). The minimum-inhibitory concentration (MIC) of vancomycin for this strain was determined to be 4 µg/mL (data not shown).

### Endolysin XZ.700

Endolysin XZ.700 was supplied by Micreos Human Health b.v. (Bilthoven, The Netherlands), and diluted to a solution of 250 mg/mL in phosphate-buffered saline (PBS, containing NaCl 8.0 g/L, KCl 0.2 g/L, Na_2_HPO_4_ 1.0 g/L, and KH_2_PO_4_ 0.2 g/L) with the addition of 0.1% bovine serum albumin (BSA; Sigma-Aldrich, St. Louis, MO, USA). Stocks were stored at −20°C until needed and diluted in PBS containing 0.1% BSA as appropriate. Test concentrations of XZ.700 were based on previous work (28).

### Collection of human saliva

Human saliva was used as a relevant condition representing the oral cavity. Saliva was collected from a single volunteer, as described previously (34) and approved by ACTA Ethics Committee ETC number 2021-40362. When appropriate, human saliva was cleared of buccal cells by centrifugation (15 min, 5,000 × *g*) and filter-sterilized using 0.22-µm filters. Saliva was aliquoted and stored at −80°C.

### Adhesion of *S. aureus* to hyphae of *C. albicans*

Adhesion of MRSA to hyphae of *C. albicans* was established as described previously (15; 35). Briefly, the Bioflux system was primed with 50% fetal bovine serum in PBS (FBS:PBS) and incubated for 30 min to coat the channels. Subsequently, the FBS:PBS was washed out with YNBG, inoculated with *C. albicans* SC5314 (set to OD_600_ of 0.3 in YNBG), and yeast cells were allowed to adhere for 30 min at 37°C under static conditions. Non-adherent cells were flushed out, and formation of hyphae was induced in YNBG at 37°C for 2.5 h. Subsequently, the *S. aureus* USA300-GFP (OD_600_≈ 0.2 in ½ TSB, 0.25% glucose (TSBG), and 10 µg/mL tetracycline) was flowed over the hyphae for 30 min to allow adhesion. The flow was stopped, and treatment was started by allowing XZ.700 solutions to flow through the channels at 0.5 dyne/cm^2^. Brightfield and FITC fluorescent images were taken every 2 min for 30 min at 20× magnification.

### Image analysis

Captured images were combined and processed into a stack using Fiji software (https://imagej.net/) (36). Brightness was auto-corrected, and the different channels representing the different colors were merged into a composite. These stacks were cropped to the relevant region, saved as TIFF, and are provided as raw data. A time ticker was added; no other modifications/image manipulations were done. Stacks were also saved as AVI using seven images/s in JPEG compression. In addition to the provided image sequence (movie), the effect of treatment was (semi)-quantified using FIJI software. Using the Time Series Analyzer plugin, the effectiveness of treatments was determined by evaluating the loss of total green fluorescence intensity (related to *S. aureus* presence) over time.

### Lytic activity assay

To determine whether XZ.700 had lytic activity against *S. aureus* USA300-GFP under representative oral conditions, XZ.700 (500 µg/mL) was prepared in FBS:PBS and 2-fold serially diluted in FBS:PBS till a concentration of 7.81 µg/mL was reached. Of each concentration, 10 µL XZ.700 was added to each row of a black, clear-bottom microtiter plate (Greiner Bio-One).

Next, cleared saliva was diluted in PBS to the following concentrations: 100%, 50%, and 25% with a final volume of 2 mL. As a 0% saliva concentration, 2 mL PBS was used. Subsequently, a 16-h *S. aureus* USA300-GFP culture was concentrated eight times by centrifugation (5 min, 5,000 × *g*), and the pellet was resuspended in 500 µL PBS. To each saliva concentration, 100 µL of the concentrated *S. aureus* USA300-GFP suspension was added, representing approximately 10^8^ CFU/mL . Each individual saliva:*S.aureus* suspension was mixed, and 90 µL of each suspension was added to each well of two columns containing the different concentrations of XZ.700 in FBS:PBS and incubated for 5 min.

The lytic effect of XZ.700 and its effect on the GFP signal were assessed, in duplicate, by determining both the optical density (OD_600_) and the fluorescence (λ_ex_/ l_em_ , 488/515 nm).

### Murine model for oral co-infection

All animal experiments were performed in accordance with the KU Leuven animal care guidelines and were approved by the Ethics Committee of the KU Leuven (project number P184/2017). Female C57BL/6J mice of approximately 9 weeks old were obtained from Janvier. Animals were housed in groups of 3 or 4 per cage. A day/night regime of 12 h/12 h was applied, and the temperature and humidity were monitored and kept constant. Cages were enriched with cotton pads, wood sticks, and paper rolls, and animals received food and water *ad libitum*.

**Fig 6:**
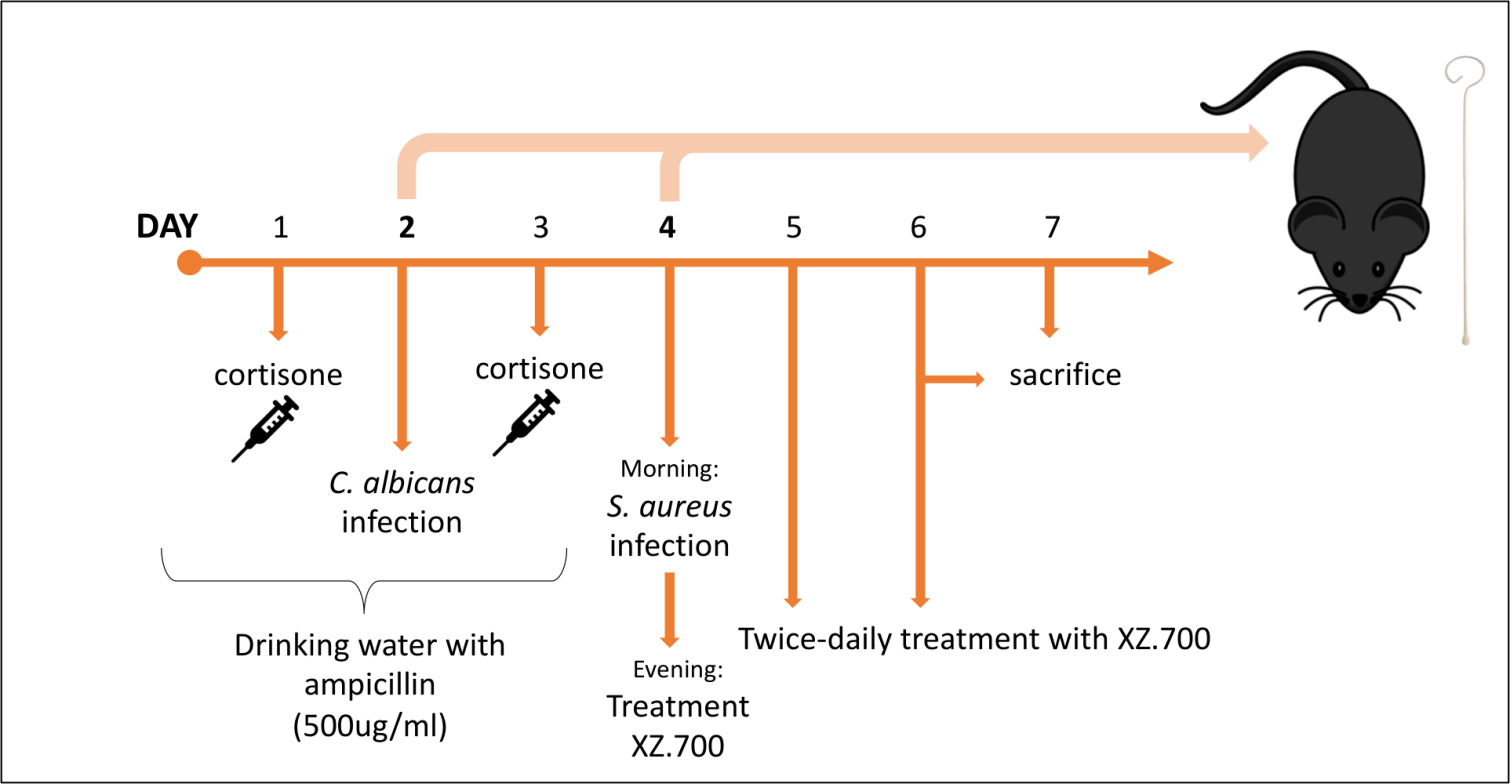
Schematic overview of the murine model for oral co-colonization with *C. albicans* and *S. aureus***.** Treatments with XZ.700 and placebo (where appropriate) were performed as described in the text.

An overview of the murine model is depicted in Fig. 6 and was previously described elsewhere (16). Mice received 0.5 mg/mL ampicillin in their drinking water 2 days before *C. albicans* infection to suppress the oral bacterial flora and allow *C. albicans* colonization. A subcutaneous administration of cortisone acetate (200 mg/kg bodyweight; ACROS Organics) was administered in the dorsum of the neck on day 1 and day 3 to render the mice susceptible to oral candidiasis. On day 2, mice were orally infected with *C. albicans. C. albicans* cells were washed twice with PBS after overnight growth in YPD, and cells were resuspended at a concentration of 1 × 10^7^ cells/mL in PBS. Calcium alginate swabs (Puritan) were saturated for 10 min in the *C. albicans* cell suspension. A maximum of 10 swabs were used per mL of cell suspension. An intraperitoneal injection of 50 mg/kg ketamine and 0.65 mg/kg medetomidine in sterile saline was used to anesthetize the mice. The calcium alginate swabs were placed sublingually for 1 h. During the infection period, mice were kept on heating pads at 37°C, and ophthalmic ointment was applied to reduce discomfort. The anesthesia was reversed by an intraperitoneal injection of 0.5 mg/kg atipamezole in sterile saline. On day 4, mice were infected with *S. aureus* in the same way. *S. aureus* cells were washed twice with PBS after overnight growth and resuspended at a concentration of 4 × 10^7^ cells/mL in PBS.

XZ.700 was administered in the drinking water at concentrations of 25 µg/mL or 100 µg/mL. Treatment started on day 4, approximately 6 h after *S. aureus* infection. The water including XZ.700 was changed twice daily, in the morning and in the evening, until sacrifice. Alternatively, XZ.700 was administered orally via a gel formulation with concentrations of 25 µg/mL and 100 µg/mL. Therefore, 20 µL of gel was pipetted directly into the oral cavity. Treatment also started on day 4, approximately 6 h after *S. aureus* infection, and was repeated twice daily until sacrifice.

Mice were monitored daily for signs of pain or decreased welfare, such as ruffled fur, hunched posture, and reduced activity. In addition, mice were weighed daily and sacrificed by cervical dislocation on day 7 or earlier if a weight loss of 20% of the total weight was observed. The tongue and kidneys were harvested in PBS, weighed, homogenized, and sonicated for 10 min in a water bath sonicator. Ten-fold dilutions were prepared and plated on bacterial and yeast chromogenic media (CHROMagar Staph aureus and CHROMagar Candida) for the enumeration of colony-forming units (CFU). When no colonies were observed in the plated dilutions, the entire remaining undiluted sample was plated. The volume of the undiluted sample available for plating varied between samples; therefore, values reported as “0 = no bacteria detected” indicate that bacterial counts were below the assay’s limit of detection and may not represent the absolute absence of bacteria.

Statistical analysis was performed using a one-way analysis of variance (ANOVA) with Bonferroni correction (*, *P* < 0.05; **, *P* < 0.01; ***, *P* < 0.001; and ****, *P* < 0.0001). The graphs display the mean and the standard error of the mean (SEM).

## SUPPLEMENTAL MATERIAL

Fig. S1 aac.01812-25-s0001.docx GFP loss upon XZ.700 treatment but not vancomycin treatment.

ASM does not own the copyrights to Supplemental Material that may be linked to, or accessed through, an article. The authors have granted ASM a non-exclusive, world-wide license to publish the Supplemental Material files. Please contact the corresponding author directly for reuse.
