# Supplementary material for: Phage-derived endolysin prevents dissemination of methicillin resistant Staphylococcus aureus during oral co-infection with Candida albicans
Source: Antimicrob Agents Chemother. 2026 Jun 15;70(8):e01812-25. doi: 10.1128/aac.01812-25 (PMC13436349; doi:10.1128/aac.01812-25)
Supplement: Supplementary file 1 [file aac.01812-25-s0001.docx]

**Supplementary information**

**Figure S1.** Loss of GFP-related fluorescence of a cell suspension of GFP-expressing MRSA over time upon treatment with XZ.700 (12.5 µg/ml), Vancomycin (4µg/ml) and a combination of XZ.700 and Vancomycin (resp. 12.5 and 4 µg/ml). For more details on loss of GFP fluorescence due to lysis of bacteria see Hoogenkamp *et al.* (33).
